# Hypophosphatemia after hemodialysis and its association with some clinical complications in patients with chronic kidney disease

**DOI:** 10.22088/cjim.13.3.527

**Published:** 2022

**Authors:** Mohammadreza Ardalan, Ali Safaei, Audrey Tolouian, Ramin Tolouian, Vahideh Ebrahimzadeh Attari, Mahsa Jalili

**Affiliations:** 1 Kidney Research Center, Tabriz University of Medical Sciences, Tabriz, Iran; 2 The University of Texas at El Paso School of Nursing, El Paso Texas, USA; 3 Division of Nephrology, University of Arizona College of Medicine, Tucson, Arizona, USA; 4 Department of Nutrition and Food Sciences, Maragheh University of Medical Sciences, Maragheh, Iran; 5 Department of Biology, Norwegian University of Science and Technology (NTNU), Trondheim, Norway

## Abstract

**Background:**

Beyond the adverse effects of hyperphosphatemia in patients with chronic kidney disease (CKD(, hypophosphatemia has also been proposed as a common challenge after dialysis. Therefore, the present study aimed to evaluate the serum phosphate level immediately after hemodialysis (HD) and its association with some clinical complications in CKD patients.

**Methods:**

The present cross-sectional study was conducted on 54 eligible CKD patients undergoing regular hemodialysis. Blood samples were taken, prior to the start and immediately after the end of hemodialysis to determine the serum levels of urea, creatinine, sodium, potassium, phosphorus, PTH, blood sugar and albumin. Moreover, the clinical complications of patients including muscle cramps, nausea, vomiting, headache, confusion, weakness and inability to speak are assessed by a questionnaire, before and after HD.

**Results:**

As we expected, the mean of serum creatinine, urea and phosphate levels significantly decreased after dialysis. Post-dialysis hypophosphatemia was graded as mild (3.5 > P ≥ 2.5 mg/dl), moderate (2.5 > P ≥ 1 mg/dl), and severe (<1 mg/dl) based on serum phosphate levels. The frequency of mild and moderate hypophosphatemia was 39.2% and 45.1 %, respectively. None of the participants had severe hypophosphatemia and 13.7% had normal phosphate levels. There was a significant correlation between post-dialysis hypophosphatemia and incidence of nausea and confusion after adjusting for confounding factors.

**Conclusion:**

To our knowledge, this is the first time that the possible association of some of the post-dialysis clinical complications with hypophosphatemia was investigated. Future large-scale studies are required to confirm the association of post-dialysis hypophosphatemia with clinical complications.

Hyperphosphatemia is the most common laboratory finding among patients with end stage renal disease (ESRD). It has been shown that hyperphosphatemia has a strong correlation with mortality and morbidity rate in chronic kidney disease. The prominent long-term consequences of insufficient phosphate monitoring are cardiovascular calcification, secondary hyperparathyroidism and metabolic bone disease (1, 2). The recommended serum phosphate level in hemodialysis patients is 3.5-5 mg/dl (3) and phosphorus levels above 6.5 mg/dl are associated with increased mortality rate (4, 5). However, hypophosphatemia has recently been proposed as a new challenge in post hemodialysis (6-8). Some case reports have already reported hypophosphatemia in patients undergoing continuous outpatient hemodialysis or peritoneal dialysis and patients admitted to the intensive care unit (ICU) due to acute renal failure (6-12).

Hypophosphatemia has also been reported in patients undergoing nocturnal hemodialysis (13). In some cases, it may require supplementation with phosphate-enriched solutions ^12^^, ^^14^^-^^16^. Hypophosphatemia is often classified as mild (3.5 > P ≥ 2.5 mg/dl), moderate (2.5 > P ≥ 1 mg/dl), and severe (<1 mg / dl) (17). Mild to moderate hypophosphatemia is generally asymptomatic while, severe hypophosphatemia is associated with severe complications like rhabdomyolysis, impaired bone mineralization, respiratory failure, central nervous system dysfunction and hemolytic anemia (18, 19).

Inorganic phosphorus acts as a small molecular weight toxin. However, the intradialytic phosphate removal kinetics significantly differ from the urea kinetics. During the first half of HD, the intradialytic drop in phosphate is quick and then declines to about 40% of pre-dialysis values. In the second half of hemodialysis, serum phosphate does not further decline, but even slightly increases towards the end of dialysis while phosphate removal continues (20, 21).

Given that some clinical symptoms such as muscle cramps, weakness and lethargy, dizziness, headache, nausea and vomiting are common after hemodialysis sessions, it is assumed that they may be associated with hypophosphatemia. Therefore, the present study aimed to evaluate the serum phosphate level immediately after HD and its association with urea removal based on Kt/V calculation and some post dialysis complications in patients with chronic kidney failure.

## Methods

**Study design and subjects:** The present cross-sectional study was conducted on patients with CKD undergoing hemodialysis in Imam Reza Hospital (Tabriz, Iran). The inclusion criteria were age ≥ 18 years, patients with 3 hemodialysis sessions per week, and the urine output less than 500 ml/day. The exclusion criteria were incidence of serious viral infections (e.g., HIV, CMV, and HBV) and parathyroidectomy. All patients were on phosphate binders' treatment including calcium carbonate and Sevelamer hydrochloride. A total of 54 eligible patients enrolled voluntarily and the informed consent forms were taken from the patients prior to the study. The Ethics Committee of Tabriz University of Medical Sciences approved this study (ethical code; IR.TBZMED.REC.1396.707). 

**Hemodialysis: **Patients received regular hemodialysis with arterio-venous fistula (AVF), three times per week (4 hours per each session). The average ultrafiltration volumes during each session of dialysis were between 2-2.5 liter. Patients were dialyzed by high flux-high efficiency biocompatible capillary filter, polysulfone (F60 or F70 filters, surface area 1.2-1.4 m^2^) and using the dialysis machine, 4008S (Fresenius Medical Care Company, Germany).

**Biochemical analysis and clinical assessments: **Blood samples were taken via the venous line of dialysis set, prior to the start and immediately after the end of hemodialysis to determine serum levels of urea, creatinine, sodium, potassium, phosphorus, PTH, blood sugar and albumin. KT/V calculations and measurements of patients' blood pressure were also performed. Moreover, the clinical complications including muscle cramps, nausea, vomiting, headache, confusion, weakness and inability to speak were assessed by a questionnaire, before and after HD.

**Statistical analysis: **Data were analyzed using SPSS software, Version 21.0 (IBM Corp., Armonk, NY, USA). The normal distribution of variables was tested by the Kolmogorov–Smirnov test, and also considering the mean and SD. Differences of biochemical parameters and clinical symptoms before and after dialysis were assessed by paired sample t- test and McNemar test, respectively. The association of serum phosphate level with KT/V and serum urea was assessed by linear regression. Moreover, logistic regression was used to examine the correlation of clinical complications (cramp, headache, nausea, vomiting, confusion, weakness and aphasia) with serum phosphate level considering adjustment for the confounding factors including age, blood sugar, serum albumin, sodium, and potassium. 

The association of clinical complications with level of hypophosphatemia after dialysis was also determined by the non-parametric Spearman's Rho test. Results are reported as mean±SD, if not otherwise stated. The significance level was set at *p *<0.05.

## Results

The baseline characteristics of the participants are shown in table 1. Accordingly, the mean of age was 58.62±16.33 years and 51% of patients were women and 49% were men. Chronic glomerulonephritis, hypertension, diabetes, and autosomal dominant polycystic kidney disease (ADPKD) were the main ESRD etiology in the participants. 

As expected, the mean of serum creatinine, urea and phosphate level significantly decreased after dialysis (*p*<0.0001) (table 2). The mean of KT/V was 1.14±0.31. As it was shown in figure 1, there was a positive significant linear association between before and after dialysis phosphate level (P=0.002) with the regression coefficient of 0.239 and the mean difference of phosphate level was -2.57±1.64 mg/dl. 

**Table 1:** Baseline characteristics of the participants (n=51)

	**Mean±SD**
Age	58.62±16.33
Sex^*^	
women	51%
men	49%
Weight (kg)	73.34±14.68
SBP (mmHg)	126.88±21.38
DBP (mmHg)	76.48±11.90
HR	74.65±21.25
BS (mg/dl)	132.38±51.39
Serum albumin (g/dl)	3.92±0.45
Serum Na (mEq/L)	138.94±2.67
Serum K (mEq/L)	4.37±0.5
KT/V	1.14±0.31

* Frequency SBP: Systolic Blood Pressure DBP: Diastolic Blood Pressure HR: Heart rate BS: Blood sugar Na: Sodium K: Potassium KT/V: Solute removal during hemodialysis

Furthermore, there was a positive significant linear association between before dialysis phosphate level and KT/V (P=0.026), but not with after dialysis phosphate level (P=0.775) and phosphate differences (P= 0.066) as it was shown in figure 2. Post dialysis serum phosphate level was graded to mild (3.5 > P ≥ 2.5 mg/dl), moderate (2.5 > P ≥ 1 mg/dl), and severe (<1 mg/dl) hypophosphatemia. The frequency of mild and moderate hypophosphatemia was 39.2% and 45.1 %, respectively. 

None of the participants had severe hypophosphatemia and 13.7% had normal phosphate level. The assessment of clinical complications of hypophosphatemia before and after dialysis showed that there were significant episodes of cramp (*p*<0.0001), headache (*p*<0.0001) and confusion (*P*=0.021) aligned with the reduction of phosphate level after dialysis (table 3). According to the findings of logistic regression test (table 4), there was a significant correlation between post dialysis serum phosphate level with nausea (*P*=0.024), and confusion (*P*=0.008) after adjusting for the confounding factors including age, blood sugar, serum albumin, sodium and potassium. Other symptoms did not indicate a significant correlation with serum phosphate level (*p*>0.05). 

Moreover, we assessed the correlation of those symptoms with graded hypophosphatemia through Spearman's Rho test. As it was shown in table 5, the results showed a significant correlation of graded hypophosphatemia with nausea (*P*=0.024) and confusion (*P*=0.005).

**Table 2:** Changes of some biochemical parameters before and after dialysis

	**Before**	**After**	**P-value**	**Mean diff (95% CI)**
Serum Urea (mg/dl)^*^	109.62 ± 39.34	41.78± 13.76	*p*<0.0001^**^	-67.84 (-76.33, -59.34)
Serum creatinine (mg/dl)^*^	6.51± 2.10	3.28 ± 1.19	*p*<0.0001^**^	-3.26 (-3.67, -2.85)
Serum Phosphate (mg/dl)^*^	5.24±1.83	2.72± 1.00	*p*<0.0001^**^	-2.57 (-3.04, -2.11)
PTH (pg/ml)^*^	347.32±231.30	271.06±202.17	*p*<0.0001^**^	-76.25 (-121.47, -31.02)

*Values are mean SD ^**^ P-values are based on the paired *t*- test

**Table 3:** Changes of hypophosphatemia related clinical symptoms before and after dialysis

	**Before**		**After**		P-value*
	**Yes (%)**	**No (%)**	**Yes (%)**	**No (%)**	
Cramp	5.9	94.1	45.1	54.9	*p*<0.0001
Headache	3.9	96.1	35.3	64.7	*p*<0.0001
Nausea	3.9	96.1	13.7	86.3	0.180
Vomiting	2	98	3.9	96.1	1.00
Confusion	2	98	17.6	82.4	0.021
Weakness	3.9	96.1	18.8	81.2	0.512
Aphasia	2	98	3	97	0.311

*P-values are based on the McNemar test

**Table 4:** Correlation of post dialysis serum phosphate level with hypophosphatemia related clinical symptoms

**Clinical symptoms**	**OR (95% CI)**	***P-*** value*
Cramp	0.75 (0.13 - 4.11)	0.743
Headache	0.65 (0.31 - 1.38)	0.269
Nausea	0.04 (0.003 -0.66)	0.024
Vomiting	1.015 (0.957-1.075)	0.625
Confusion	0.023 (0.001 - 0.375)	0.008
Weakness	0.345 ( 0.080 - 1.500)	0.156
Aphasia	0.315 (0.35- 2.805)	0.300

*P-values are based on the logistic regression

**Table 5:** Frequency of positive clinical symptoms in relation with graded serum phosphate level after dialysis

	**P ≥ 3.5 mg/dl**	**3.5 > P ≥ 2.5 mg/dl**	**2.5 > P ≥ 1 mg/dl**	***P-*** value*
Cramp	8.7%	39.1%	52.2%	0.322
Headache	11.1%	50.0%	38.9%	0.655
Nausea	0.0%	14.3%	85.7%	0.024
Vomiting	14.6%	41.7%	43.8%	0.146
Confusion	0.0%	11.1%	88.9%	0.005
Weakness	11.1%	33.3%	55.6%	0.549
Aphasia	14.3%	40.8%	44.9%	0.311

*P-values are based on the Spearman's Rho test

## Discussion

The results of the present study confirmed a statistical decrease in serum phosphorous (P) and urea concentration immediately after HD. Interestingly; the Kt/V based on urea clearance did not correlate with the level of the serum phosphate. According to our data, patients with higher P levels at pre-dialysis had higher P levels post-dialysis. The frequency of mild and moderate hypophosphatemia was 39.2% and 45.1 %, respectively in this study. None of the participants had severe hypophosphatemia and 13.7% had normal serum phosphate level. Similar to our finding, post-dialysis hypophosphatemia was previously reported in patients under continuous dialysis and patients with acute renal failure (ARF) admitted to ICU (6-12, 16).

Although, we did not measure the total phosphate removal, Elias et al. reported that total phosphate removal is higher in those with higher level of hyperphosphatemia^22^.The compartment effects are important during hemodialysis and the dialyzer can only remove the exposed molecules. Urea has a multi-compartmental nature, but it can move easily across the cell membranes; therefore, its compartmentalization is less pronounced in hemodialysis. Phosphorous has a multi compartmental nature, but its inter-compartmental mass transfer is assumed to differ from that of urea, as their molecular characteristics are different. During dialysis, fractional removal of urea mass drops with the prolonged dialysis time due to a diminished concentration gradient. In contrast, the diffusion gradient for phosphate does not decline, leading to a continued inorganic phosphorous mass removal (23-25). A longer dialysis time rises the removal of phosphate mass even when Kt/V is not changed. A possible reason might be that approximately 90% of total body phosphorus is intracellular and distributed in the soft tissues and bones, and only 1% is stored in the interstitial spaces. Phosphorous kinetics during HD have been known at least as a 2-phase model. In the first stage that takes 1.5-2 hours, the reduction of phosphate is mainly due to its extracellular compartment removal. In the second stage that usually occurs when pre-dialysis phosphate levels dropped to 40-50%, most of the phosphorous removal comes from the intracellular compartments and bones (26). Although during the first hour of dialysis, the most phosphorous is removed, the continuous removal happens during the later stages of hemodialysis. Each four-hour standard hemodialysis session can remove approximately 600 to 1200 mg of phosphate^21^. A multi-compartment model might be more suitable to explain the biological manner of phosphate removal (27). Moreover, we examined the prevalence of post-dialysis symptoms and to our knowledge, this is the first time that possible association of some of the post-dialysis clinical complications with hypophosphatemia were investigated. Our results showed that there is a significant correlation between post dialysis serum phosphate level and nausea and confusion. However, other symptoms did not have significant correlations with serum phosphate level. Beyond the various side effects of hyperphosphatemia, severe hypophosphatemia is associated with some complications like rhabdomyolysis, impaired bone mineralization, respiratory failure and central nervous system dysfunction. 

Moreover, mild to moderate hypophosphatemia can be accompanied by minor complications like lethargy and dizziness ^18^^-^^19^. A recent case report by Koganti and Sam has shown that two hemodialysis patients showed the symptoms of encephalopathy as a severe post dialysis hypophosphatemia. The symptoms had been reversed when phosphorus was added to the dialysate solution baths (12). As a limitation of our study, the intradialytic phosphate kinetics or phosphate rebound which occurs few hours after dialysis were not assessed. Moreover, the other limitations of this study are lack of nutritional assessment of patients and the small sample size of study. 

In conclusion, given the evidence of hypophosphatemia after chronic or continuous outpatient dialysis and patients with ARF admitted to the ICU, it is recommended that this issue can be considered in hemodialysis sessions and if there are clinical symptoms of hypophosphatemia, phosphorus-containing dialysis solutions can be used to prevent irreversible complications. Future large-scale studies are required to confirm the association of post dialysis hypophosphatemia with clinical complications.
